# Laser-Scribed Graphene for Human Health Monitoring: From Biophysical Sensing to Biochemical Sensing

**DOI:** 10.3390/nano14110942

**Published:** 2024-05-27

**Authors:** Yakang Li, Yaxin Li, Sirui Wu, Xuewen Wu, Jian Shu

**Affiliations:** 1 Hunan Institute of Advanced Sensing and Information Technology, Xiangtan University, Xiangtan 411105, China; 2 Department of Chemical Engineering, School of Chemical Engineering, Xiangtan University, Xiangtan 411105, China

**Keywords:** laser-scribed graphene, health monitoring, biophysical sensing, biochemical sensing, flexible sensor

## Abstract

Laser-scribed graphene (LSG), a classic three-dimensional porous carbon nanomaterial, is directly fabricated by laser irradiation of substrate materials. Benefiting from its excellent electrical and mechanical properties, along with flexible and simple preparation process, LSG has played a significant role in the field of flexible sensors. This review provides an overview of the critical factors in fabrication, and methods for enhancing the functionality of LSG. It also highlights progress and trends in LSG-based sensors for monitoring physiological indicators, with an emphasis on device fabrication, signal transduction, and sensing characteristics. Finally, we offer insights into the current challenges and future prospects of LSG-based sensors for health monitoring and disease diagnosis.

## 1. Introduction

Human health is a fundamental cornerstone of societal development, and maintaining a sound state of physical and mental well-being is crucial not only for an individual’s quality of life but also as an essential enabler of sustainable economic and social progress. However, factors such as population aging, lifestyle changes, and environmental pollution have led to a rising incidence of chronic and emerging diseases [1]. Coupled with the stressful living and working conditions of modern society, human health faces unprecedented challenges. Consequently, comprehensive, timely, and accurate monitoring and diagnosis of individual health statuses have become pressing needs. Traditionally, disease diagnosis and health monitoring have relied on professional medical institutions like hospitals and clinics, which offer face-to-face consultations, examinations, and regular physical check-ups. While this model has achieved significant milestones over the past decades, it exhibits several limitations in addressing the current and future healthcare needs amid challenges such as an aging population, the rising prevalence of chronic diseases, and increasing global health demands. These limitations include uneven resource distribution, poor accessibility, a lack of timely and continuous health monitoring, and difficulties in implementing personalized treatments. In response, there is a growing need to develop advanced health monitoring technologies, now a critical research focus in the biomedical field. Flexible wearable sensing, as an emerging technology in recent years, has offered new approaches to address these pressing health challenges [2]. The development of wearable and flexible sensors strongly depends on the advances of material science, and innovative nanomaterials with exceptional properties are being actively explored to meet the growing demand for disease diagnosis and health monitoring. Among them, graphene stands out as a promising candidate due to its unique characteristics.

Graphene, a two-dimensional material composed of a single layer of carbon atoms arranged in a hexagonal lattice via sp^2^ hybridized orbitals, is recognized as one of the most promising carbon-based materials and has attracted significant attention [3]. Due to its sensitivity to both chemical and biological molecules, graphene is an ideal material for sensor design. It boasts a high specific surface area (2630 m^2^/g), excellent electrical conductivity (approximately 1.0 × 10^8^ S/m), and ultrahigh electron mobility (200,000 cm^2^/V/s). Additionally, the superior mechanical strength (1 TPa), thermal conductivity (2000–5000 W/m/K), high transparency (97.4%), and biocompatibility position it as a prime candidate for developing flexible electronic devices [4]. Despite its potential, the widespread practical application of graphene still faces significant challenges, including material preparation, device patterning, and functional modifications. Traditional methods of graphene preparation, such as chemical vapor deposition (CVD) and mechanical exfoliation, while capable of producing high-quality graphene films, are complex, costly, and struggle with achieving uniform production over large areas. Wet chemical methods, involving a series of oxidation and reduction steps, washing, and centrifugation, are energy-intensive and result in considerable reagent consumption and pollution. Furthermore, graphene obtained through these methods typically contains a high level of defects and impurities. Additionally, precise etching processes make patterning graphene a significant technical challenge. These bottlenecks limit the adoption of graphene materials in health monitoring applications.

In this context, the discovery and development of laser-scribed graphene (LSG) have opened new avenues for the preparation and application of graphene. By precisely controlling laser parameters, it is possible to directly form patterned three-dimensional porous graphene structures on various common carbon-containing substrates, such as polymer films and fibers. This mask-free, pollution-free, and scalable manufacturing process effectively addresses the bottlenecks in graphene preparation and patterning technologies [5]. It is important to note that porous carbon structures formed by laser irradiating carbon-containing precursors are often referred to as LSG, laser-engraved graphene (LEG), laser-derived graphene (LDG), or laser-induced graphene (LIG), although they do not strictly consist of a single or a few layers of sp^2^-carbon lattice. Given that laser scribing is pivotal in both the formation and patterning of these materials, we describe all these patterned carbon structures derived from polymeric and other natural carbon sources as LSG in this review. The annual increase in studies on LSG-based sensors for human health monitoring highlights their growing importance and emergence as a research hotspot. To date, several key reviews have reported on the advancements of LSG, with a focus on fabrication, engineering, and their promising applications in various electronic devices [6,7,8,9,10,11,12]. As a supplement of previous summaries and updates, this review briefly introduces the technical principles and advantages of LSG and summarizes the current research status of LSG-based sensing, particularly emphasizing the physical and biochemical indicators and their recent breakthroughs in health monitoring. Finally, a concise analysis of the current technical limitations and challenges of LSG-based sensors for health monitoring and promising directions are presented. This review does not aim to cover all developments in LSG-based sensing; instead, it highlights the key aspects and significant advancements of the research that are considered most relevant. Due to the diversity of techniques, extensive research across broad application areas, and our own limitations in scope and knowledge, we inevitably fail to mention many important contributions. We deeply regret any unintentional exclusions and extend our apologies to the authors whose works have been overlooked and to the readers who may find their areas of interest are not covered.

## 2. Key Influencing Factors to Properties of LSG

LSG is a porous graphene structure formed on the surface of specific carbon source materials through direct laser irradiation. The laser-scribing technique has shown advantages such as being a single-step, mask-free, cost-effective, and reagentless fabrication method compared to the conventional fabrication methods of graphene-based devices. Although the specific formation mechanism of LSG is controversial and depends on the characteristics of the laser sources and the carbon precursors, most types of lasers produce a photothermal effect, where two key processes, pyrolysis and carbonization, are primarily involved. Due to the gas release and thermal expansion during the formation of LSG, the surface and interior of the material spontaneously form a porous 3D network structure, which greatly increases the specific surface area and endows LSG with unique advantages in many fields such as sensing, energy storage, and catalysis.

### 2.1. Carbon Precursor Species

The chemical structure and thermal stability of carbon precursors are key factors in determining the effectiveness of LSG preparation. These factors not only affect the generation efficiency of LSG but also influence its properties. For example, PI film can be converted to LSG in air, while transforming pine wood into hierarchical porous graphene requires a reducing or inert gas atmosphere, likely due to the differences in thermal stability between PI and wood [13]. The inherent crosslinked lignocellulose structure in wood is essential for the formation of LSG, and the lignin content significantly impacts the quality of LSG. Currently, high-performance thermoplastic resins rich in aromatic rings such as polyimide (PI), polyether ether ketone (PEEK), and polysulfone (PSU) are preferred as LSG precursors because of their good thermal stability and appropriate light absorption rates, which make them more prone to carbonization and formation of high-quality graphene structures. Inspired by the fabrication of LSG on wood, a variety of naturally occurring lignocellulosic materials have also been successfully converted into LSG by laser induction and further processed to form biodegradable devices. However, operational parameters must be clearly adjusted based on the laser source and the properties of different carbon precursors. Thus, the absence of a universal tool for producing LSG with various carbon precursors presents a barrier to the low-cost, large-scale production of LSG devices. In 2018, the Tour group reported a generally applicable technique, multiple pulsed-laser scribing, that can fabricate LSG on any carbon precursor capable of being converted into amorphous carbon [14].

### 2.2. Processing Parameters and Conditions

**Figure 1:**
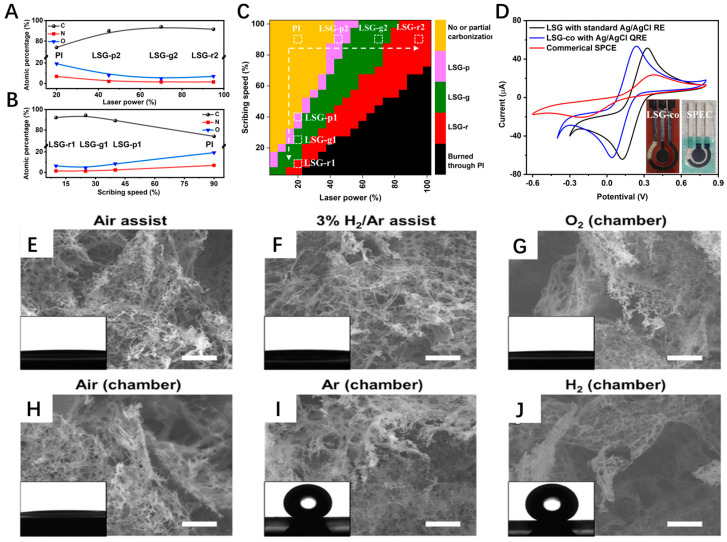
Dependence of compositions of LSG versus laser (**A**) power and (**B**) scan speed; (**C**) heatmap of LSG quality vs. laser power and scan speed; (**D**) CV curves of the optimized LSG electrode and SPCE; (**E**–**J**) SEM images and contact angles (inset) of LSG samples prepared under different gas atmospheres (Scale bars, 2 µm).

Power density, scanning rate, laser wavelength, and other parameters directly affect the microstructure, chemical composition, electronic, and mechanical properties of LSG. The scanning rate is critical as it determines the residence time of the laser beam on the precursor surface, which, in turn, influences the thickness of the LSG. A slower scanning rate facilitates the accumulation of heat, promoting the formation of thicker LSG. Additionally, the scanning rate affects the number of residual doping atoms and defects in the LSG. Laser power density has a decisive effect on the maximum temperature in the LSG formation region and significantly affects the thickness, number of layers, crystallinity, and defect density of the LSG. If the power density is too low, it may be insufficient to fully initiate the carbonization and graphitization reactions; conversely, excessively high power densities can lead to severe pyrolysis and ablation. Although most previous studies on LSG have employed discrete parameters, the effectiveness of a laser-based device often stems from the synergistic interaction of multiple laser parameters. Our previous work explored the correlation between the fabricating process and electrochemical properties of LSG [15]. The experimental results demonstrated that the structure and composition of LSG were associated with vital fabrication parameters (Figure 1A,B). Laser scribing offers great process latitude for the quality optimization of LSG, and the optimal combinations of laser power and scribing speed exhibit a roughly linear correlation (Figure 1C). Appropriately reducing the laser power and scribing speed facilitates the fabrication of a high-quality LSG electrode that exhibits superior electrochemical performance compared to commercial screen-printed carbon electrodes (SPCE), as shown in Figure 1D. Pulse stacking density (pulses per inch, PPI) and lines per inch (LPI) are useful parameters for controlling the LSG structure and properties. They determine whether a sheet-like or fiber-like structure forms [16]. Depending on the wavelength, the laser sources used for LSG manufacturing can be divided into ultraviolet (<400 nm), visible light (400–750 nm), near-infrared (NIR, 0.74–2.5 μm), and medium-infrared (MIR, 2.5–25 μm) lasers. The infrared laser induces graphene formation through photon absorption and the subsequent thermal decomposition of the carbon precursors, while the ultraviolet laser achieves carbonization by directly breaking the chemical bonds. The UV/visible laser has higher energy and better induction efficiency, and the LSG it prepares usually has a narrower linewidth, which is conducive to the fabrication of micro sensors, such as micro nanoelectrodes. Compared to LSG fabricated with an infrared laser, research on the relationship between the sensing properties of LSG fabricated with UV and its morphology, comparison, and structure is rare. Santos et al. compared LSG fabricated by IR (IR LSG) and UV (UV LSG) lasers and their applications as dopamine (DA) electrochemical sensors [17]. Although it exhibits lower sensitivity at physiologically relevant DA concentrations compared to IR LSG, UV LSG is still an excellent material for DA detection because it provides a slightly higher linear range and faster heterogeneous electron transfer (HET) kinetics. Studies reveal that the UV femtosecond laser (343 nm, 220 fs) can directly fabricate LSG that possesses superior sheet resistance (10 Ω sq^−1^) on any wood or even thin leaves in an air atmosphere with negligible photothermal ablation [18].

Laser scribing performed in different gas atmospheres (air, Ar, N_2_, H_2_, etc.) influences the precursor decomposition reaction, which, in turn, affects the surface morphology, defect type, and chemical composition of LSG. For example, the hydrophobic properties of LSG surfaces can be regulated by altering the gas atmosphere [19]. Under reducing or inert environments, surfaces with superhydrophobic properties (contact angle > 150°) were obtained. LSG fabricated under an oxidizing atmosphere exhibits superhydrophilicity (~0°) (Figure 1E–J). The variation in hydrophobicity is related to the oxygen content and morphology of LSG. Reduced oxygen content on the surface of graphene and the formation of carbon nanoparticles on the surface can enhance hydrophobicity. The hydrophobicity of F-doped LSG, prepared under an SF_6_ gas atmosphere, can be further enhanced due to the low surface free energy of the C-F bonds, achieving contact angles greater than 150°.

Although some properties of LSG may be slightly inferior to graphene produced by mechanical exfoliation or CVD, the relative simplicity and cost-effectiveness are the main advantages of this technique. The structure, composition, morphology, and properties of LSG can be regulated by optimizing the laser scribing process conditions and parameters, offering potential for its application across different fields.

## 3. Modification of LSG

**Figure 2:**
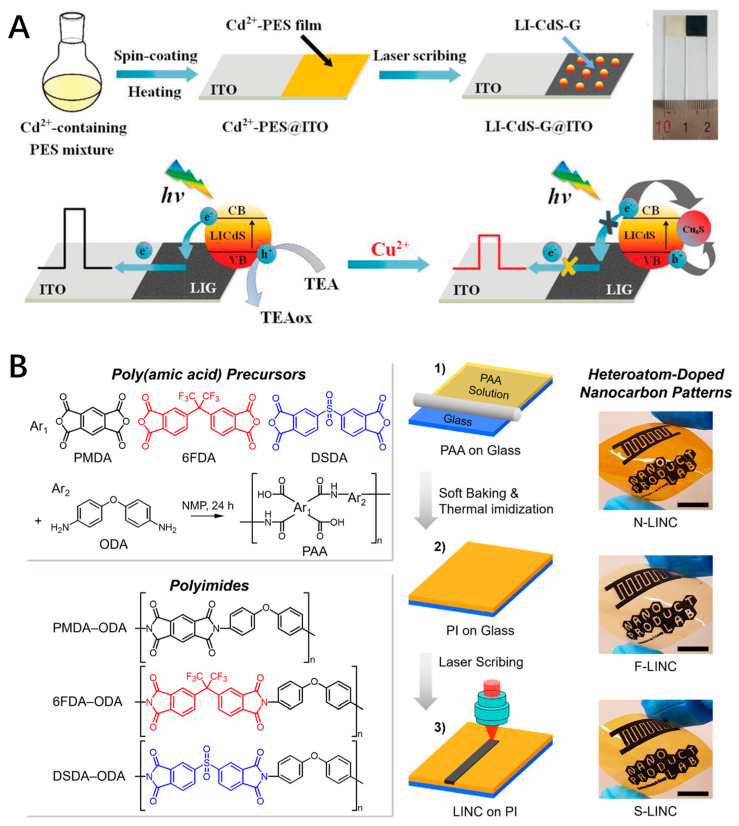
(**A**) Schematic illustration for the preparation of LSG-based composite photoelectrode; (**B**) Heteroatom-doped LSG fabricated with molecularly controlled polyimides (scale bar, 1 cm).

To enhance performance or impart specific functionalities to LSG, it can be modified and doped through various methods. The main strategies include precursor blending modification, surface modification, and elemental doping. Precursor blending involves pre-doping the polymer precursor with functional components, such as metal salts or inorganic nanoparticles. These components are then transformed into uniformly distributed functional modification phases within the LSG structure, resulting in the formation of composite LSG materials. Ye et al. developed a method to fabricate porous graphene embedded with various types of nanoparticles [20]. Initially, metal complexes are mixed with poly(pyromellitic dianhydride-co-4,4′-oxidianiline amic acid) (PAA) in an organic solvent to obtain a metal-complex-containing PI film. Subsequently, direct laser scribing of this film results in the formation of graphene embedded with various types of nanoparticles. Because the properties of this structure can be tuned by controlling the complex precursor, it represents a flexible and customizable strategy for different application requirements. Employing a similar strategy, Ge et al. successfully fabricated metal sulfide and metallic-oxide-decorated graphene nanocomposites on indium–tin oxide glass through CO_2_ laser irradiation [21,22]. These composites, serving as photoelectrodes, exhibited rapid and stable responses for photoelectrochemical sensing (Figure 2A).

Surface modification performed on pre-prepared LSG is the most common method for regulating properties and extending the functionality of LSG to meet specific application requirements. Surface modification can be achieved through various methods, including chemical modification, biomodification, physical deposition, and electrochemical modification. Chemical modifications, such as carboxylation, silanization, and amination, introduce various functional groups to enhance the hydrophilicity, hydrophobicity, or bioactivity of LSG. Biomodification involves the direct bonding of biomolecules, such as nucleic acids, enzymes, and antibodies, to the LSG surface, enhancing its applications in the biomedical field. Physical deposition techniques, such as atomic layer deposition and chemical vapor deposition, are used to form layers of metal nanoparticles or oxides on the LSG surface, thereby improving its electrochemical and photoelectrical properties. Electrochemical modification involves the electrodeposition of conductive polymers or metals and their oxides onto the LSG surface, primarily to enhance its performance in electrocatalysis, energy storage, and sensor devices.

In addition to the inherent structural properties of polymers, the precursor can also be doped with other elements or compounds, thus, introducing dopant atoms into LSG and endowing it with unique chemical and electronic properties. As demonstrated in Figure 2B, Nam et al. synthesized three viscous poly(amic acid) (PAA) intermediate solutions with different backbones through a two-step polycondensation process of 4,4-oxydianiline (ODA) with three different tetracarboxylic dianhydrides (PMDA, 6FDA, and DSDA) [23]. They fabricated N, F, and S-doped porous graphene microelectrodes on the corresponding polyimides using a continuous-wave CO_2_ infrared laser. This work demonstrates the effectiveness of controlling the heteroatom composition of LSG by adjusting the molecular structure of the PI without the need for external doping sources such as gas atmospheres or additive molecules. The obtained F-doped LSG microelectrodes, featuring a high surface area, achieve nanomolar-level detection of dopamine and demonstrate a three-orders-of-magnitude improvement in sensitivity compared to carbon fiber microelectrodes, the gold standard in electrochemical dopamine sensing. To date, substituting carbon atoms with heteroatoms such as B, N, P, S, and F is an important method that can tailor the electronic band structure, introduce electrochemically active sites, and enhance the electron transfer process. Wan et al. reported that laser scribing of PI induces N-doping (2.4−4.5%) in the graphene skeleton in the forms of pyrrolic N and graphitic N, which improves its conductivity and affinity for nucleic acids in biosensing [24]. Additionally, LSG can be doped with other elements by coating carbon precursors with specific components. For example, thermally pressing a fluorinated ethylene propylene (FEP) film (10 μm) onto a PI film (50 μm) forms a composite film as the carbon precursor [25]. By exploiting the extremely different absorption rates of FEP and PI films to a UV laser (355 nm), F-doped LSG was fabricated by a one-step interfacial laser-induced method on the composite film. Due to the doping with F and the specific microstructures, the F-doped LSG exhibits superhydrophobic properties.

## 4. LEG-Based Biophysical Sensing

Owing to their distinct electronic and mechanical properties, LSG and its derivatives are promising candidates for monitoring biophysical parameters and play important roles in a wide range of applications, from personal healthcare to human–machine biointerfaces [26]. For instance, minute changes in physiological signals such as pulse wave, joint movement, respiratory rate, and body temperature can be continuously monitored in real time, providing early warnings for the risk of conditions such as hypertension, arthritis, and respiratory diseases. In this section, we summarize the advancements in flexible LSG-based sensors for monitoring vital signs.

### 4.1. Electrophysiological Monitoring

**Figure 3:**
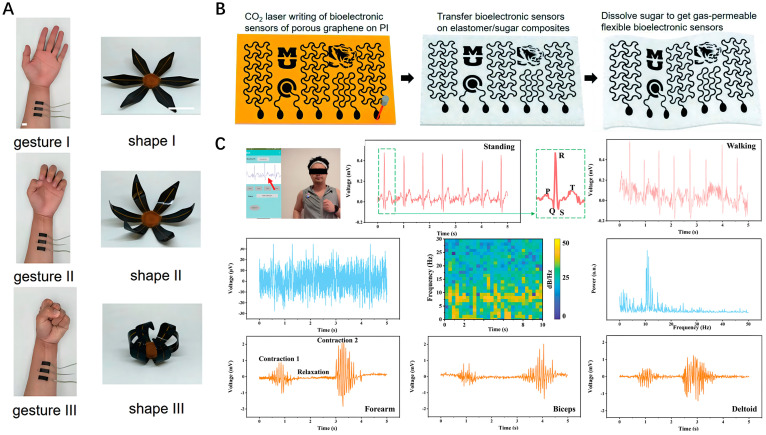
(**A**) Demonstrations of human-soft actuator interaction via real-time EMG monitoring; (**B**) Manufacturing of gas-permeable, multifunctional on-skin bioelectronic sensing systems using porous materials; (**C**) On-body monitoring of ECG, EEG, and EMG using the LSG-based sensory system.

In both resting and active states, biological cells or tissues of organisms generate regular electrical activities, which are termed bioelectrical signals. The fundamental mechanism behind these bioelectrical signals involves the transmembrane flow of ions, encompassing both the resting potential and the action potential. Human bioelectrical signals contain an abundance of physiological characteristic information. The research on electrophysiological signals can advance our understanding of fundamental human physiological activities, characterize health conditions, or serve as assessment tools in clinical medical treatment. Clinical bioelectric signals typically include electrocardiography (ECG), electroencephalography (EEG), electromyography (EMG), and electrooculography (EOG). Due to these signals having high contact impedance, low strength, and significant common-mode noise, high-performance electrodes are crucial for accurate signal acquisition. In the early stages, the monitoring of biopotential signals was primarily conducted using rigid electrodes. However, long-term attachment to the skin can cause discomfort, and such electrodes struggle to maintain stable conformal contact with the skin. For biopotential signal monitoring, this can introduce motion artifacts, thereby compromising the reliability of the target data collected. Therefore, bioelectrodes should possess a wide dynamic range, low impedance, high precision and signal-to-noise ratio, good durability, and excellent stability within a range of deformation. Graphene and its derivatives are well-suited to meet the performance requirements of bioelectrodes and are widely used in the collection of bioelectrical signals and have achieved good results. For example, employing CO_2_ laser patterning of LSG on a commercial PI film, Ling et al. fabricated soft electrothermal actuators. Human gestures can control the 3D structure of the flower-shaped LSG-based actuator through real-time EMG signals recorded by three LSG-based EMG sensors (Figure 3A) [27]. Usually, most LSG devices are directly fabricated on PI substrates, which is one of the limiting factors for ensuring gas permeability and long-term practicability. Fabricating flexible devices using materials with high breathability and biocompatibility is beneficial to form a compliant skin contact, which improves the quality of the detection signals and the comfort of attachment. Abd-Elbaki et al. employed a CO_2_-pulsed laser to fabricate highly conductive LSG-SF electrodes from low-cost and non-conductive silk fabric (SF) [28]. These electrodes feature a uniform conductive layer on both sides, enhanced by silk fibroin/Ca^2+^ for better skin adhesion and antibacterial effectiveness. This advancement led to LSG-SF electrodes with lower impedance and superior stability in electrophysiological signal recording compared to traditional Ag/AgCl electrodes. The experimental results demonstrated that these LSG-SF electrodes are particularly effective in capturing consistent electrocardiogram signals during extensive physical activity. Using carbon-containing porous materials as substrates to directly fabricate gas-permeable on-skin sensors is an effective strategy to ensure comfort. Artificially introducing a porous structure into existing functional materials is also an effective strategy to tailor gas permeability, stretchability, and modulus of LSG devices. Figure 3B exhibits a typical procedure to fabricate gas-permeable, multifunctional LSG-based sensing devices on porous elastomer sponges [29]. First, various LSG-based bioelectronic sensors are patterned on a PI substrate via CO_2_ laser. Then, these patterns of porous graphene are transferred onto partially cured composite substrates composed of soft silicone elastomer and cane sugar powders. The fabrication process is completed by curing the samples and dissolving the sugar using deionized water. The ECG and EMG signals measured by gas-permeable LSG-based sensors can match those recorded with conventional Ag/AgCl gel electrodes, demonstrating exceptional proficiency in capturing electrophysiological activities from the skin. Additionally, the exploration of flexible integrated sensing systems has also aroused widespread research interest. Zhang et al. have developed a three-in-one portable electronic sensory system based on a low-impedance (<100 Ω) LSG on-skin electrode [30]. This compact sensory system integrates wireless communication, power supply modules, and a mobile application and is capable of simultaneously monitoring EEG, ECG, and EMG signals in real-time. This cost-efficient and versatile electronic sensory system paves the way for advances in wearable health monitoring (Figure 3C).

### 4.2. Motion Monitoring

The recent developments in flexible sensors, employing various sensing mechanisms, have made significant progress in the field of human motion monitoring. These sensors can accurately capture essential signals from different body parts during routine activities or specific movements. Thus, they offer data support ranging from daily health monitoring to complex clinical diagnoses. Human motion monitoring covers the entire spectrum from large-scale movements like the bending of knees, hands, and fingers to small-scale movements such as chest respiration and swallowing actions. These mechanical stimulations, generated from body movements, can be monitored by tensile and pressure sensors.

**Figure 4:**
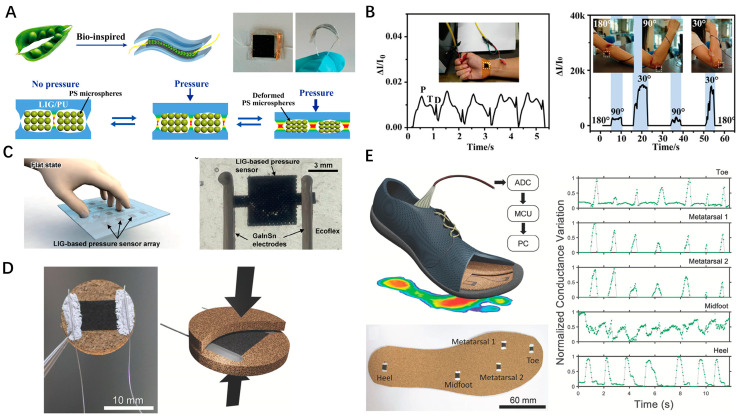
(**A**) Schematic of the bean pod-inspired flexible pressure sensor and (**B**) its application in monitoring blood pulse and elbow bending; (**C**) Schematic image of a stretchable tactile pressure sensor array and the photo of a sensor; (**D**) LSG-on-cork piezoresistive compression sensors; (**E**) LSG-on-cork piezoresistive insole monitoring of human gait.

Flexible pressure sensors primarily rely on variations in electrode spacing or contact area between electrode materials and dielectrics under pressure, which alter their electrical characteristics. They are widely used in the biomedical field to monitor key health indicators such as intraocular pressure, heart rate, blood pressure, gait, and more. A diverse range of materials has been employed for pressure sensing using a similar mechanism. Compared to traditional materials, LSG, with its inherent three-dimensional porous structure, often exhibits superior piezoresistive properties and sensitivity to mechanical stimuli, along with flexible manufacturing capabilities. For example, Huang et al. introduced a flexible capacitive pressure sensor (FCPS) by fabricating LSG on a PI film and subsequently transferring it onto a porous PDMS foam [31]. The high porosity of the PDMS foam allows more significant deformation under certain pressure loadings, enabling the FCPS to achieve high sensitivity and fast response. It has been successfully applied to detect joint movements, body pressure, and arterial pulse. Tian et al. introduced a novel pressure sensor inspired by the bean pod structure, featuring a microspacer core layer of polystyrene (PS) microspheres sandwiched between two LSG/polyurethane (PU) films (Figure 4A) [32]. The porous 3D interconnected network structure of LSG offers numerous cavities for the PS microspheres. The microspheres act as spacers to regulate the number of contact points and the contact area at each point upon compression, hence, altering the conductance and improving sensitivity. This flexible pressure sensor has been successfully applied in monitoring human pulse and limb motion (Figure 4B). To comfortably cover surfaces on 3D objects, flexible sensors should maintain stretchability while retaining their sensitivity and precision. However, stretching sensors often impact their stability in output. To address this issue, Li et al. proposed a stretchable tactile pressure sensor array using LSG as the sensing material, liquid metal alloy (GaInSn) as electrodes, and an elastic ecoflex polymer as a protective layer (Figure 4C) [33]. Processed by machine learning algorithms, this system accurately monitors tactile pressure under various stretching states and adeptly recognizes actions. Although CO_2_ lasers are commonly utilized in LSG production, the research indicates that laser sources with shorter wavelengths, such as visible and UV lasers, typically provide higher resolution and the capacity for patterning on thinner substrates [34]. Carvalho et al. innovatively produced LSG on natural cork by 355 nm UV laser irradiation [35]. Assembling the LSG pattern with an unprocessed cork sheet, the piezoresistive response was continuously recorded by connecting the two short edges of the LSG pattern to a measurement device (Figure 4D). Applying compressive pressure on the porous structure of the LSG results in resistance variation. The proposed piezoresistive sensors show high sensitivity (0.02 kPa^−1^), fast response (0.5 ms), and a broad sensing range (28–1132 kPa), enabling precise monitoring of human gait through the analysis of pressure signal variations across various regions of the foot during different gait phases (Figure 4E).

**Figure 5:**
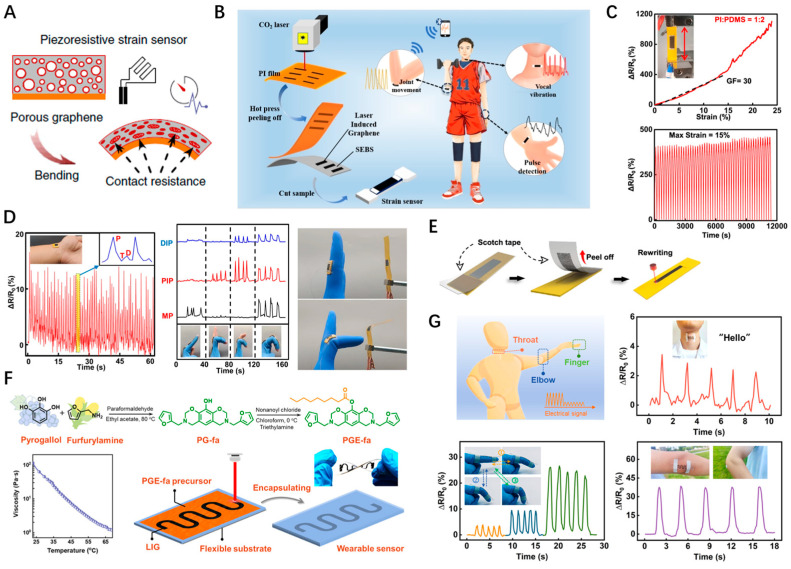
(**A**) Mechanisms of LEG-based strain sensing; (**B**) Flexible LSG-based strain sensor fabricated by heat transfer printing techniques for health monitoring; (**C**) The stretchability and recyclability of the LSG-based strain sensor; (**D**) LSG-based strain sensor monitoring human pulse rate waves, finger motion, and as a tool to remotely control the bending motion of actuators; (**E**) Schematic illustration of the rewriting procedure of LSG on PI/PDMS composite substrate; (**F**) Schematic diagram of PGE-fa synthesis and fabrication of LSG-based sensors; (**G**) Monitoring of human motion in real time.

In daily life, one of the key functions of skin is to perceive external forces and produce strain. Inspired by this, people have been striving to develop flexible strain sensors that can mimic the deformation sensing capabilities of human skin. Key performance indicators for these sensors include high sensitivity, exceptional stretchability, and durability, among others. Unlike traditional strain gauges that have limited working ranges and sensitivity, LSG-based strain sensors exhibit a wider range and greater sensitivity, largely due to their three-dimensional porous structure. The application of external strain compresses this structure, resulting in a significant change in resistance (Figure 5A) [36]. Additionally, a unique sensing mechanism arises from the displacement of graphene layers, leading to changes in interlayer contact resistance. These features make LSG-based sensors an ideal choice for wearable applications [37,38]. Most studies involve the preparation of LSG on PI or aromatic-rich polymer substrates. However, the tough and rigid plastic substrates pose constraints on their applications in fields that require high-level stretchability. A common solution is to infiltrate the open porous structure of LSG with a liquid polymer precursor, followed by heat curing to transfer the LSG to a solid elastomer substrate such as PU, PDMS, or Ecoflex, thus, expanding the applicable area of the flexible strain sensor [39,40,41]. Although the composite materials provide the superior mechanical stability required for strain sensing, the elastomer covering part of the LSG surface inevitably impairs its performance as a sensing electrode. Recently, Liu et al. observed plenty of carbon vacancies in the crystalline lattice of LSG by examining the microstructure of atom-layer thin LSG flakes [42]. These atom-level configured defects may induce fractures or delamination within LSG sponges. Leveraging this property, they transferred LSG foams to solid elastomer substrates via heat transfer printing techniques and developed a flexible LSG-based strain sensor (Figure 5B). This sensor exhibits outstanding electromechanical characteristics, such as extraordinary sensitivity (gauge factor: 413–3118), negligible hysteresis, and a broad strain range (>100%). It enables real-time monitoring of human body movements, such as skin expansion and muscle contraction, integrated with a wireless communication module. Wang et al. proposed soft and stretchable electronics fabricated on a PI/PDMS composite with laser scribing [43]. The PI/PDMS composite substrate was prepared by mixing PI particles with a PDMS precursor, where the PI particles serve as the carbon source for LSG, and PDMS provides flexibility and stretchability. The LSG-based sensors can fit complex 3D configurations and exhibit a 470% linear response and good cyclability under a maximum strain of 15% (Figure 5C). The applications of the LSG-based sensor for monitoring human pulse rate waves and finger motion, and as a tool to remotely control the bending motion of actuators, were demonstrated (Figure 5D). Notably, the LSG sensor manufactured on the PI/PDMS composite substrate is erasable and rewritable, which improves the utilization efficiency of the substrate, as illustrated in Figure 5E. Liu et al. developed a direct conversion of a liquid organic precursor into versatile 3D graphene by rapid laser irradiation, providing a promising strategy for the facile and transfer-free fabrication of stretchable graphene-based devices [44]. Recently, they synthesized a biobased benzoxazine liquid precursor (PGE-fa) that could be coated on various flexible substrates such as PDMS, PET, and paper and transformed into graphene by laser irradiation (Figure 5F) [45]. The flexible LSG-based strain sensor with optimized structural design, obtained from the transfer-free strategy, has achieved a wide working range (∼30%), high sensitivity (GF = 68,238.5), long-term stability (10,000 cycles), and rapid response (∼0.3 s) as well as recovery (∼0.35 s). These sensors, capable of detecting a range of external stimuli at different bending angles, have proven their versatility in monitoring human movements, from minor activities involving the vocal cords to limb motion of fingers and elbow joints (Figure 5G).

### 4.3. Body Temperature Monitoring

**Figure 6:**
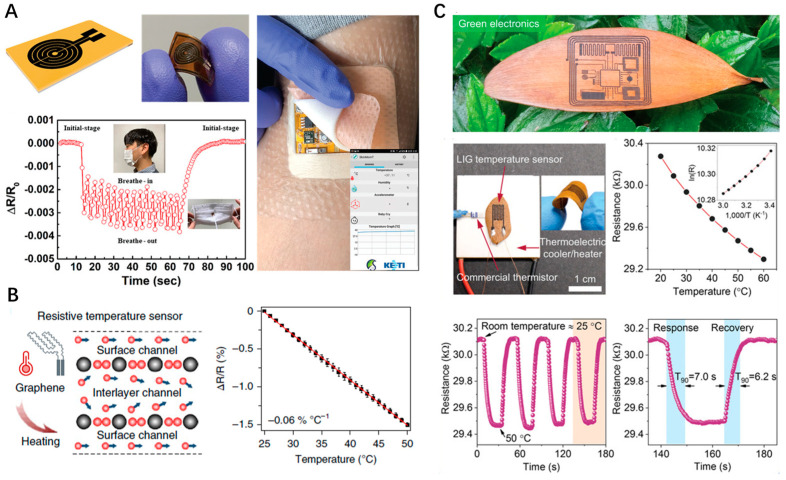
(**A**) LSG-based temperature sensors integrated with flexible printed circuit boards for breath monitoring; (**B**) Mechanism of LEG-based temperature sensing and linear response characteristic; (**C**) A flexible electronic containing a temperature sensor fabricated on a leaf and its temperature sensing performance.

Temperature is one of the most commonly perceived pieces of information in nature. From a physiological perspective, body temperature is closely linked to health conditions. However, people generally measure their body temperature only when feeling unwell or when they believe it is necessary. The importance of accurately monitoring human body temperature in real-time stems from its critical role in reflecting metabolic levels, assessing patient recovery, and aiding athletes in training and competition scheduling. Flexible temperature sensing devices have received increasing attention in recent years. Recent advancements, such as the development of LSG-based temperature sensors integrated with flexible printed circuit boards, highlight innovation in personal health monitoring (Figure 6A) [46]. The patterned LSG exhibits a linear response (R^2^ = 0.999) in the temperature range of −10 to 60 °C, with a negative temperature coefficient of resistance (−0.142%/°C), and can monitor health-related activities such as breathing. The high resistance of 200 kΩ endows the sensor with low power consumption, and it has been applied in a wearable patch-based device to transmit body temperature information to smartphones in real-time. The superior electrical response of LSG-based temperature sensors originates from electron–phonon scattering and the thermal velocity of electrons in the graphene layers (Figure 6B) [36]. Zhang et al. comprehensively explored the influence of laser power density and scan path width on the linearity and sensitivity of LSG-based temperature sensors on a PI substrate [47]. They found that the optimal parameters for laser power density and path width are 76 J/cm^2^ and 0.8 mm, respectively. The sensor showed excellent linearity (R^2^ = 0.999), sensitivity (0.05%/°C), and negligible hysteresis within a temperature range of 30 °C to 100 °C. The sensor possesses excellent mechanical properties and retains the ability to continuously measure temperature even after being bent 500 times. Compared to polymers, woods and their derivatives, such as cellulose, are considered the most common green materials, being biodegradable, sustainable, and abundant on earth. High-performance flexible temperature sensors can also be fabricated from these low-cost natural materials. Le et al. reported the one-step generation of patterned graphene with high conductivity on arbitrary woods and leaves using ultrafast high-photon-energy laser pulses (FsLDW). A flexible electronic containing LSG electrical interconnects, a temperature sensor, and a pseudocapacitor was fabricated on a leaf (Figure 6C) [18]. The temperature coefficient of resistance for the sensor fabricated on a leaf was assessed to be −0.08%/°C, and the response and recovery times were 7.0 and 6.2 s in the range of 25–50 °C, respectively. The authors also explored the influence of substrates on the sensing performance. They found that the sensor fabricated on wood exhibited a similar temperature coefficient, but the response (27.2 s) and recovery (75.4 s) were markedly slower. This difference results from the substrate structure. Leaves, with their much thinner thickness (0.1 mm), possess more efficient thermal transfer compared to wood (thickness, 2 mm).

## 5. LEG-Based Biochemical Sensing

**Figure 7:**
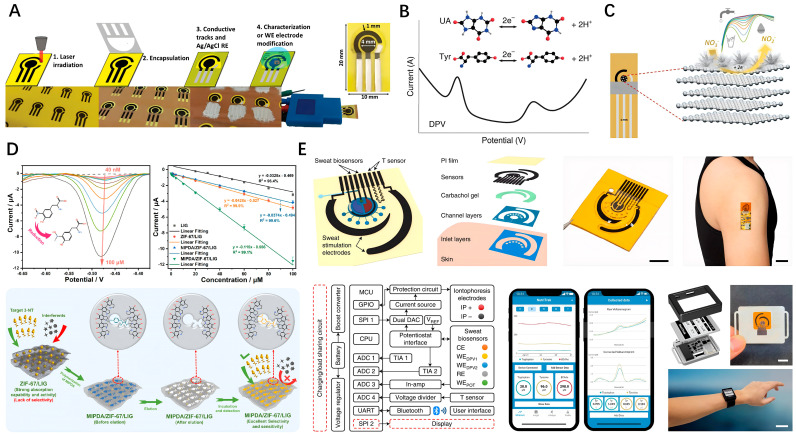
(**A**) Schematic representation of fabrication workflow of LSG three-electrode systems; (**B**) Simultaneous detection of UA and Tyr with voltammetric methodologies; (**C**) Electrodeposited silver on LSG for electrochemical detection of nitrate; (**D**) Detection performance and mechanism of the MIPDA/ZIF-67/LIG microsensor for the 3-NT; (**E**) A wearable LSG-based electrochemical biosensing system for the monitoring of metabolites and nutrients. (scale bar, 5 mm).

Biosensors are devices that convert biochemical or biological reactions into physicochemical signals for the quantitative analysis of targets, providing crucial solutions for evaluating human health at the molecular level. They are also extensively applied in food and drug safety analysis, environmental monitoring, and public safety, playing a significant role in modern life. LSG, with its high specific surface area, excellent electrochemical activity, good biocompatibility, and facile fabrication process, demonstrates substantial potential in the field of biosensing. Electrochemical sensors represent the most widely explored class of LSG-based sensors; the typical fabrication is illustrated in Figure 7A [48]. In fact, graphene-based electrodes have long been used for electrochemical sensing. However, conventional methods of graphene preparation (such as graphene oxide reduction, mechanical exfoliation, CVD) and patterning (including photolithography, transfer, inkjet printing, and screen printing) have notable deficiencies and limitations. Moreover, the introduction of additives, fillers, and polymeric binders in the construction of graphene electrodes inevitably impairs the charge transfer rates and electrical conductivity and reduces the specific surface area of the electrodes. Thus, manufacturing graphene-based electrochemical sensors while mitigating these detrimental effects is crucial for sensing applications.

Fortunately, LSG is prepared and patterned by a reagentless process that effectively avoids the use of additives and binders and exhibits mechanical and chemical robustness. Additionally, the porous structure of LSG provides numerous active sites and a high surface area. The research has shown that the heterogeneous electron transfer (HET) rate constant (k^0^) of an LSG electrode (0.1150 cm/s) for potassium ferrocyanide(II) is significantly higher than those of similar carbon-based materials, such as edge plane pyrolytic graphite (0.022 cm/s) and basal plane pyrolytic graphite (10^−9^ cm/s) [49]. This advantage is attributed to the binder-free 3D porous network of LSG with enriched edge plane sites.

Benefiting from the abundant catalytic active sites resulting from the laser irradiation process and the excellent electrochemical stability, bare LSG electrodes can be directly used for detecting a variety of electroactive molecules. For instance, conductive porous carbon electrodes were produced on paperboard using a CO_2_ laser, achieving an active-to-geometric area ratio of 6.5. These electrodes have been successfully used for detecting potassium ferricyanide, ascorbic acid, caffeic acid, and picric acid using different electrochemical technologies [50]. Employing voltammetric methodologies, bare LSG electrodes can also be used to simultaneously distinguish and quantify multiple electroactive molecules with intrinsically distinct redox potentials in a mixed system. Yang et al. developed an entirely laser-engraved sensor capable of continuous sweat sampling, detection of temperature, respiration rate, and low concentrations of uric acid and tyrosine [36]. The levels of uric acid and tyrosine are important indicators for diseases such as gout and metabolic disorders and can be assessed through their respective oxidation peak current values (Figure 7B). Introducing doping elements or decorating LSG with metallic nanoparticles, polymers, and other nanomaterials typically endows it with new sensing mechanisms and improved properties, such as conductivity, electrocatalytic activity, electron transfer kinetics, and selectivity [51]. Electrodepositing Pt nanoparticles (Pt NPs) on LSG electrodes can increase the electrochemical active surface area by 13.14% [49]. Additionally, the k^0^ value for [Fe(CN)_6_]^4−^ increases from 0.1150 to 0.2823 cm/s, and for [Ru(NH_3_)_6_]^3+^, it rises from 0.0868 to 0.2312 cm/s. Due to significantly enhanced electrocatalytic oxidation activity, the sensitivities of the LSG electrodes deposited with Pt NPs for detecting ascorbic acid, dopamine, and uric acid increase from 237.76 to 250.69 μA mM^−1^ cm^−2^, 2259.9 to 6995.6 μA mM^−1^ cm^−2^, and 5405 to 8289 μA mM^−1^ cm^−2^, respectively. Adiraju and colleagues developed an LSG electrode modified with silver dendrites to address the issue of nitrite interference during the detection of nitrate in acidic and neutral media (Figure 7C) [52]. Samoson et al. fabricated an LSG–chitosan–gold nanoparticle electrode by a one-step laser radiation process on a PI film [53]. This composite electrode, with excellent electrical conductivity and electrocatalytic activity toward the oxidation of uric acid, enables on-site monitoring of uric acid in blood serum by coupling to a portable potentiostat connected to a cellphone.

Despite their excellent electrochemical properties, these electrodes still face significant challenges in achieving specific detection in complex systems. In fact, prior knowledge of the system under study is essential for successful analysis with negligible interference. However, it is challenging in practical detection to determine the presence of electroactive species and their impact on the analyte. Furthermore, the detection being limited to only electroactive molecules severely restricts the application of electrochemical sensing techniques. To overcome these limitations, LSG is often modified with surface elements that have a strong affinity for the analyte. For example, the incorporation of ion-selective membranes into LSG enables real-time potentiometric detection of various ions, such as H^+^, Na^+^, K^+^, NH_4_^+^, Cl^−^, and Ca^2+^. Because human electrolyte ion values are generally maintained within a stable range, these devices can be used for noninvasive personal healthcare, sports management, and disease diagnosis through precise monitoring of ions in body fluids [54,55,56].

Molecularly imprinted polymers are also widely used to functionalize LSG electrodes to improve selectivity and sensitivity [57,58,59]. Polymerization occurs in the presence of the analyte, with subsequent elution or dissociation by appropriate methods to form cavities that specifically bind the analyte in samples. Zheng and colleagues modified LSG with ZIF-67 and subsequently crafted molecularly imprinted polydopamine using dopamine as the functional monomer and L-tyrosine as the dummy template [60]. Benefiting from the synergy of MIPDA, ZIF-67, and LSG, this imprinted electrode was used for detecting 3-nitrotyrosine, exhibiting excellent sensitivity with a detection limit of 6.71 nM and distinguished selectivity against 11 interfering substances (Figure 7D). Wang et al. developed an electrochemical biosensing system that contains LSG-based sensing electrodes, an iontophoresis-based sweat induction module, a microfluidic sweat sampling module, a signal processing and calibration module, and a wireless communication module (Figure 7E) [61]. By functionalizing LSG electrodes with metabolite-specific antibody-like molecularly imprinted polymers and redox-active reporter nanoparticles, the electrochemical biosensor achieved continuous analysis of trace levels of metabolites and nutrients in sweat, including all essential amino acids and vitamins.

**Figure 8:**
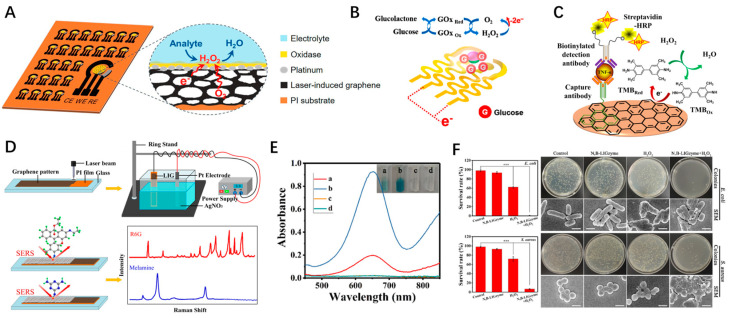
(**A**) Schematic illustration of the LSG-based enzyme electrode and sensing mechanisms for (**B**) glucose detection and (**C**) typical electrochemical immunoassay; (**D**) Fabrication and application of the AgNPs@LIG SERS chip; (**E**) Evaluating peroxidase-like activities of different materials via absorption spectra; (**F**) Bactericidal effects of N,B-codoped LSG (Asterisks indicate statistically significant differences, *** *p* < 0.001. Scale bars, 1.0 μm).

Employing biomolecules such as enzymes, antibodies, and aptamers as recognition elements is a prominent strategy due to two major characteristic advantages. First, the specific interaction between the recognition element and the target molecule confers high sensitivity and specificity, even within complex systems. LSG-based electrochemical enzymatic biosensors have garnered widespread attention for their excellent specificity and capability for real-time and continuous monitoring. The reaction occurs between the substrate molecule and the immobilized enzyme, facilitating electron exchange with the LSG electrode and generating a measurable current signal (Figure 8A) [62]. LSG serves as an ideal carrier material because its porous 3D interconnected network structure enhances electron transfer behavior by providing a large surface area and shortening the diffusion distance of substrate molecules within the interior of the electrode. The typical sensing principle of a glucose biosensor is illustrated in Figure 8B [63]. It exhibited a high sensitivity (69.64 μA mM^−1^ cm^−2^) within a detection range of 5–3000 μM (covering the glucose range in sweat) and a low limit of detection (LOD) (0.23 μM). Employing a similar strategy, LSG electrodes have also been effectively utilized for the selective detection of other important small molecules including uric acid, ascorbic acid, hydrogen peroxide, and cholesterol. Secondly, by ingeniously designing recognition elements and sensing strategies, these sensors can quantify a diverse range of targets, including ions, small molecules, proteins, nucleic acids, cells, and viruses [64]. For example, electrochemical immunosensing is usually designed based on enzyme-catalyzed reactions, as illustrated in Figure 8C [15]. In 2019, the global pandemic of SARS-CoV-2 posed a significant challenge to public health, which drove the development of inexpensive and rapid diagnostic devices. Using LSG electrodes modified with capture antigens and antibodies respectively, Gao’s group reported a novel multiplex, portable, and wireless electrochemical platform for the ultra-rapid detection of viral antigen nucleocapsid protein, IgM and IgG antibodies, and C-reactive protein [65]. In 2021, Beduk et al. used electrodeposited gold nanostructures on LSG to develop a similar electrochemical immunosensor for the quantification of SARS-CoV-2 levels in blood serum [66]. The accuracy and applicability of the proposed sensor were verified by a clinical study.

Here, we primarily review the applications of LSG in electrochemical sensors. In fact, LSG has also shown promise in the fields of electrical and optical biosensing. For instance, to address the challenges of uniformity and repeatability in ultra-sensitive detection of trace targets in SERS sensing, Han et al. developed an AgNPs@LIG SERS chip using PI as a substrate (Figure 8D) [67]. By assembling AgNPs on porous LSG through electroplating to enhance the local electromagnetic field, and using rhodamine 6G as a probe molecule, this SERS chip achieved a limit of detection (LOD) of 10^−14^ M and an enhancement factor of 3 × 10^10^ with good uniformity (relative standard deviation about 9.6%). It achieved the nanomolar-level detection of melamine, an illegal food additive. Chen et al. found that LSG with N-doping exhibits inherent and specific peroxidase-mimicking nanozyme activity and can catalyze the chromogenic reaction of 3,3′,5,5′-tetramethylbenzidine, the classical chromogenic reactions used in an enzyme-linked immunoassay (Figure 8E) [68]. A more than 4.5-fold enhancement of peroxidase-like activities was achieved by loading H_3_BO_3_, a boron source, to form N,B-codoped LSG. Thus, it is expected to be used in chromogenic biosensing applications. Additionally, the experimental results demonstrated that N,B-codoped LSG catalyzes the decomposition of H_2_O_2_, generating highly active hydroxyl radicals (Figure 8F). It exhibits strong bactericidal properties against both Gram-negative (Escherichia coli) and Gram-positive (Staphylococcus aureus) bacteria, even at low levels of H_2_O_2_.

## 6. LSG-Based Multi-Modal Sensing Integration

**Figure 9:**
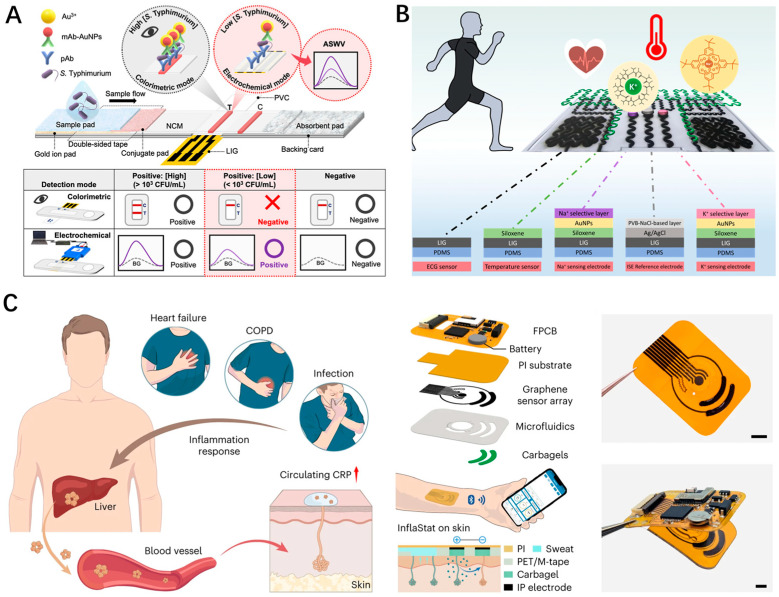
(**A**) Schematic illustration of dual colorimetric/electrochemical detection of salmonella typhimurium using a LSG–LFIA strip; (**B**) Functionalization of LSG-based sensing electrodes of the hybrid patch and application diagram; (**C**) Schematics of the wireless LSG-based patch for the monitoring of C-reactive protein in sweat (scale bars, 0.5 cm).

Because the human body is a highly complex system, accurately evaluating human health often necessitates the precise monitoring of specific indicators on one hand and comprehensive analysis of various physiological parameters on the other. However, rapidly detecting trace analytes in biological samples accurately and cost-effectively remains challenging due to background interference from complex substrates. A promising approach to overcome this challenge is to employ a dual-mode sensing strategy that utilizes independent signal readouts based on two different response mechanisms. This method not only leverages the complementary strengths of each mode but also enables the mutual confirmation of detection results obtained through different modes, thus, enhancing the reliability of the outcomes. A typical example is shown in Figure 9A [69]. A LSG-electrode-integrated lateral flow immunoassay (LIG-LFIA) strip was developed for the sensitive and rapid detection of Salmonella enterica serovar Typhimurium with colorimetric and electrochemical dual modes. The colorimetric signal, acquired from an AuNP-labeled monoclonal antibody, and the electrochemical response, acquired from the electrodeposition of Au^3+^, could be utilized for early screening and high-sensitivity quantitative analysis, respectively.

Integrating sensing modes for various biophysical and biochemical indicators on a single wearable platform is essential for the comprehensive, accurate, and continuous monitoring of human physiological states [70]. As a multifunctional sensing material, LSG is favored for the synchronous acquisition of multi-mode signals due to its design flexibility. Recently, some integrated sensor arrays have been successfully developed for real-time monitoring of a wide range of physiological parameters. For example, Hui et al. developed an electrochemical–physiological (Chem-Phys) multifunctional LSG-based patch for simultaneously monitoring sweat electrolytes, temperature, and ECG (Figure 9B) [71]. The device is expected to overcome the reported problem that the T-wave shape of the ECG signal may be affected by the potassium concentration in the human body. Another ingenious work worth mentioning is from Tu et al., who developed a wearable and wireless patch for the accurate monitoring of C-reactive protein in sweat based on the LSG-based flexible sensor array, as schematically illustrated in Figure 9C [72]. The sensor array consists of an anti-CRP capture antibody-modified electrode, an ionic strength sensing electrode, a polyaniline-modified pH sensing electrode, and a temperature sensing electrode. Thus, the dynamic information of pH, ionic strength, and skin temperature can be simultaneously monitored and used for real-time CRP sensor calibration, which can provide more comprehensive and accurate health data. Through the construction of a big data analysis model, the massive amount of physiological data from LSG multimodal sensors will be deeply mined for intelligent analysis. This will facilitate more accurate disease warnings, health assessments, and personalized medicine, thereby promoting innovation and development in healthcare. It can be anticipated that as an integrated, low-cost intelligent sensing solution, LSG will excel in the fields of telemedicine monitoring, health warning, and vital sign analysis.

## 7. Summary and Outlook

As a new type of three-dimensional porous carbon nanomaterial, LSG shows great application potential and broad development prospect by virtue of its special structure and excellent electrical properties, particularly in the field of human health monitoring. A timely and inspiring review on these aspects is valuable, promoting further improvement in this emerging research field. This paper summarizes the latest research progress of LSG in the field of health monitoring and disease diagnosis. The influence of key processing parameters, and modification treatment technology are briefly introduced. The innovative applications and technical characteristics of the LSG-based sensor in physical signals such as pressure, strain and temperature and biochemical signals such as electrolyte ions, metabolic small molecules, and protein macromolecules are comprehensively explored.

Although significant progress has been made in the field of physiological signal monitoring with LSG, several challenges still need to be addressed. Current reports shown that key indicators such as stability, sensitivity, selectivity, and response speed of LSG-based sensors require further optimization to meet the stringent demands of practical applications. It is essential to enhance the response by tailoring LSG size, morphology, doping, defects, interfaces, and other multilevel structures, thereby comprehensively improving sensing performance. LSG is typically fabricated using a laser direct writing method, which is inefficient and limited to small areas. Future developments should focus on new large-area manufacturing processes to enable the large-scale, cost-effective production of LSG and its devices. Given the complexity of human health, the integration of multiple sensing modes into a LSG-based sensing platform is crucial. Novel device designs and integration with microelectronics, communication technologies, and big data analytics are needed to enable collaborative fusion and intelligent analysis of multimodal signals, providing a comprehensive assessment of individual health conditions and realizing the ultimate goal of precision medicine. Last but not least, further enhancements in flexibility, comfort, mechanical stability, and adaptability to complex environments like humidity and sweat are necessary for the wearable applications.

Overall, LSG holds promise as a versatile and effective material. It is anticipated that LSG flexible electronic technology will bring revolutionary changes to the fields of disease diagnosis and health monitoring and provide a new impetus for the sustainable development of human health with the unremitting efforts of the scientific and technological community and the industry.
